# Nurses’ intention to care for patients with infectious disease: a content analysis study

**DOI:** 10.1186/s12912-023-01538-9

**Published:** 2023-10-03

**Authors:** Esmaeil Hoseinzadeh, Abbas Ebadi, Tahereh Ashktorab, Hamid Sharif-Nia

**Affiliations:** 1 Department of Nursing, Faculty of Nursing and Midwifery, Tehran Medical Sciences, Islamic Azad University, Tehran, Iran; 2 Behavioral Sciences Research Center, Life Style Institute, Baqiyatallah University of Medical Sciences, Tehran, Iran; 3 Nursing Faculty, Baqiyatallah University of Medical Sciences, Tehran, Iran; 4 Traditional and Complementary Medicine Research Center, Addiction Institute, Mazandaran University of Medical Sciences, Sari, Iran; 5 Department of Nursing, Amol Faculty of Nursing and Midwifery, Mazandaran University of Medical Sciences, Sari, Iran

**Keywords:** Nurses intention, Care, Patient, Infectious disease, Content analysis

## Abstract

**Objective:**

This present study was designed to explain the concept of nurses intention to care of patients with infectious diseases.

**Methods:**

This study is a deductive content analysis study that was performed from May 2022 to Jun 2022 in three hospitals in Iran. In total 21 nurses were chosen by purposive sampling and for deta collection used semi-structured interviews. Data analysis was done using Elo and Kingas method.

**Results:**

This study have revealed the formation of seven distinct themes, namely Job satisfaction, Professional ethics, Personal values, Standard precautions, Preserving health, Support, and Attitude of patients and their families. These themes are comprised of 17 categories and 59 subcategories.

**Conclusion:**

By comprehending the dimensions of nurses’ intentions to care for patients with infectious diseases, it is possible to develop suitable planning and strategies to meet the healthcare requirements of such patients. Managers can take action by examining the issues and demands of nurses, and by providing job security, they can establish a Healthcare service systems with high security that can effectively respond during an outbreak of infectious diseases. Additionally, Nursing managers can prevent nurses from leaving their jobs by taking appropriate intervention, increasing their motivation, and enhancing their satisfaction.

**Supplementary Information:**

The online version contains supplementary material available at 10.1186/s12912-023-01538-9.

## Introduction

The provision of high-quality nursing care is essential in ensuring the well-being of patients [1]. Care, which is defined as the act of being attentive and careful towards someone or something, is a fundamental aspect of nursing practice [2]. The focus of nursing policies is on improving and ensuring the quality of care provided to patients [3]. Nursing care plays a critical role in the healthcare system, and it is vital in ensuring patient satisfaction [4, 5].

Nurses who work in infectious disease ward have a crucial role in preventing and controlling infectious diseases, and they contribute significantly to public health services [6]. However, during the crisis of infectious epidemics, nurses may experience fear and anxiety due to the severity of the disease, mortality rates, and susceptibility to infection. This can potentially affect their health, well-being, and work effectiveness. Therefore, it is essential to provide support and resources to nurses to ensure their safety and well-being while delivering high-quality care to patients [7].

Nurses’ working conditions put them at risk for occupational illnesses and injuries as well as healthcare-associated infections. deficiency of manpower, particularly nurses, is one of the basic factors which limits the ability of hospitals in confronting emerging infections [8]. Nurses are in the first line of caring and have an important role in caring, however, working in stressful circumstances and insufficient resources, lack of information about infectious diseases, Fear of getting sick and spreading the illness to their family can negatively affect their intention to care and cause to leave their jobs [9].

The nurses intention to care for infectious patients and nurses intention to leave this job, coupled with the lack of at least 100,000 nurses in Iran, have made it problematic to provide the health needs of these patients [10].

Therefore, nurses’ intention to care is crucial, and it provides an explanation and prediction of behavior as the cornerstone of intervention [11]. The term intention is defined as purposeful and prompt action. In the nursing literature, “intention” implies providing holistic nursing care with love [12]. The importance of nurses’ intention to care has been emphasized by Sir Adams and Wright, who believed that what is essential is not what nurses do but how and for what purpose they do it [13].

In conducting surveys on nurses’ experiences about caring for patients with infectious diseases, various themes have emerged. These themes include fear and uncertainty [14], adoption of leadership models for managing urgent conditions, changing nursing approaches, emotional and physical effects on nurses, and worthiness and team spirit [15]. In Iranian nurses, the experiences were related to incomplete preparation, worst perceived risk, family support, social stigma, and selfless commitment [16]. Overall, the results indicate that nurses express different experiences regarding the intention to care for patients with infectious diseases based on their cultural background, the type of infectious disease, and their personal and professional circumstances. Therefore, it is imperative to consider these factors when designing interventions aimed at enhancing nurses’ intention to provide care for patients with infectious diseases [14–16].

The intention of nurses to provide care is a complex and multifaceted concept that is influenced by a range of factors. These factors include job satisfaction, organizational commitment, the workload of nurses, satisfaction with financial compensation, experience in caring for infectious diseases, perceived behavioral control, and subjective norms [9, 17].

The diversity of these factors highlights the pivotal influence of cultural norms in each country on the intention of nurses to provide care. Given the fundamental significance of nursing and its intention to care, which are vital components in delivering high-quality nursing services for infectious diseases, it is essential to explicate nurses’ comprehension of the concept of intention to care for patients with infectious diseases.

## Methods

### Sudy design

This present studuy was content analysis study to “explain the concept of nurses’ intention to care for patients with infectious disease” that was accomplished from May 2022 to Jun 2022.

### Participants

Number of 21 nurses were interviewed by purposeful sampling method among the nurses in north east of Iran. The interviews were done in the Staff rest room and Nursing Office. The interviewes were developed for this study. We uploaded an English language version of a interview as a supplementary file.

### Data collection

At this stage of the study, various methods of semi-structured interview, observation, audio recording and writing them down on paper and taking notes during the interview were used to collect information. The initial questions included, what made you intent to care of infectious patients?Or how did you feel about these patients before facing them, and what about now? And the follow-up questions continued based on the initial answers of the participants and in order to obtain deeper information and clarify the studied concept (Table 1). Also, during the interview, using questions such as ((What do you mean? Please explain more about this? Or my impression of your words is, have I understood correctly?)) were used in order to deepen the interview. The interview time varied from 35 to 45 min and 21 interviews were conducted until data saturation was reached. When the researcher thoroughly investigates each category and identifies its various traits and dimensions under various circumstances [18].

**Table 1:** List of pre-interview open-ended and targeted questions

Open-ended Questions	Targeted Questions
Please explain how you felt about these infectious patients before encountering them, and how do you feel now? Please explain what made you intent to care of infectious patients?	What concerns do you have in caring for infectious patients? What is your opinion regarding the care of these high-risk patients in terms of disease transmission? How is your inner belief about the intention to care?

### Data analysis

To analyze the data, the deductive qualitative content analysis method of Elo and Kingas was used [19]. Also, in qualitative data analysis, MAX-QDA version 10 software was used to manage and organize data.

### Preparation phase

The preparation phase started with the selection of the unit of analysis, which was the typed text of the interviews with nurses. The written materials were reviewed numerous times so that the researcher was totally comfortable with all the data before immersing themselves in it and no insight or theory could be extracted from the data until the data was completely.

### Organization phase

In this step, open coding, creating categories and abstracting were done. Codes that were duplicated or overlapped in terms of meaning or did not conceptually explain the intention to care for infectious patients were removed. Then, the primary codes that indicated a single topic were placed in the same category based on a continuous comparative analysis between the data of each interview and the data of other interviews and paying attention to the similarities, differences and proportions. Sub-category and the maine Category were created by continuing the comparison of primary classes based on similarities, differences, and proportions. All these processes were carried out with the participation of all four authors (E.H, A.B, T.A and H.S) independently.

### Reporting phase

In this stage, the sampling process, characteristics of the participants, method of data collection and analysis, and each of the main categories were reported in detail.

### Trustworthiness of study

Terms such as credibility, conformability, dependability, transferability, conformability and authenticity describe the trustworthiness of qualitative content analysis. Trustworthiness of this study was described for all three stages of qualitative content analysis [20].

### Trustworthiness in the preparation phase

In this study, interviews and their transcripts were used for data collection, and semi-structured questions were used to lead the interviews. Interviews with nurses who had prior experience caring for patients with infectious diseases were conducted in addition to focused sampling. Sampling and interviews with nurses were done until data saturation was reached, and after each interview, transcripts were made and data analysis was done. The appropriate unit of analysis was the transcribed text from interviews with nurses.

### Trustworthiness of organization phase

In order to strengthen the study’s credibility at this point in the content analysis, the researchers used all their efforts to explain how to summarize the data and form the classes. For the purpose of verifying the findings, a researcher in charge of analysis and supervisors and advisors followed the process of analysis and classification. Also, to ensure the proper interpretation of the data and to confirm their correct analysis, the data were seen by the study participants.

### Trustworthiness of reporting phase

In reporting the content analysis of this study, the researchers attempted to provide a comprehensive description of the aspects of content analysis and culture, concepts, selection process, and participant characteristics in order to assure transferability. In order to confirm the results of the content analysis, the researchers tried to make the findings reflect the statements of the participants, and for this reason, the appropriate quotes of the participants that are related to all the concepts were reported. Also, the researchers tried to increase the validity and accuracy of the results for the research readers by fully explaining the process of analyzing and abstracting the concepts and explaining the relationship between the results.

## Results

In this study the mean age of the nurses was 32.66 years (Table 2). From the interviews with the participants, totally seven main categories were formed, which include Job satisfaction, Professional ethics, Personal values, Standard precautions, Preserving health, Support, and Attitude of patients and their families. (See details in Table 3)

**Table 2:** Demographic characteristic of nurses

Variables	% (*n*)
Age	32.66 ± 4.77
Work Experience(years)	10.14 ± 4.81
Sex Male Female	23.8(5) 76.2(16)
Marital status Single Married	33.3(7) 66.7(14)
Occupation Unemployed Employed	52.4(11) 47.6(10)
Education BS MS	90.5(19) 9.5(2)

**Table 3:** Dimensions of nurses’ intention to care for patients with infectious disease

Main Category	Generic Category	Subcategory
Job satisfaction	Belonging to the profession Accepting the nature of the job	Job satisfaction Belonging to the profession Accepting the nature of the job Job attachment Job Contentment Career Passion Awareness of the dangers of the disease Acceptance of job Hazards Job compatibility
Professional ethics	Professional commitment Professional values Understanding the patient’s situation	Responsibility Conscientiousness Sincere performance Work conscience Love for humankind Sacrifice Being a supporter Sympathy Attention to the patient’s needs Relieve the patient
Individual values	Spirituality Self-esteem	Attention to the prayer of the patient The importance of pleasing God Faith in God Feeling valuable To be effective Feeling good about the patient’s recovery Enjoy care Increasing the level of knowledge and skills
Standard precautions	Occupational safety Preventive measures	The presence of personal protective equipment Sufficient equipment Standard physical structure Compliance with the principles of control and prevention Use of personal protective equipment Constant caution
Preserving health	Health anxiety Recognition of illness Prioritizing personal and Family health physical and mental stress	Fear of contagion to the family Fear management in experienced nurses Lack of knowledge of the nature of the disease Unknown diagnosis Known diagnosis The importance of personal health The importance of family health The importance of the health of the vulnerable person in the family Special family circumstances Physical tension Mental stress Exhaustion
Support	Financial support Non-financial support	Wage increase Financial incentive Low-income level Family support Support of department colleagues Head nurse support Increasing the number of nurses Educational support Recruitment organization Media support
Attitude of patients and their families	Behavioral reflection Patient and family satisfaction	Appropriate behavioral response of the patient Inappropriate behavioral response of the patient The Behavioral reaction of the patient’s family Understanding the patient from the nurse Patient Satisfaction Satisfaction of the patient’s family

### Job satisfaction

Job satisfaction plays a very important role in a nurse’s life, so that job performance, early retirement, job transfer, organizational commitment, as well as patient safety and satisfaction are affected by this concept [21]. The sensation that a person has about her or his employment in relation to prior experiences and present expectations is known as job satisfaction [22]. An important element in keeping nurses on staff and delivering high-quality care is job satisfaction [23]. Job satisfaction in nurses refers to belonging to the profession and accepting the nature of the job.

### Belonging to the profession

According to the statements of the nurses, Job attachment, job happiness, and career enthusiasm are the things that lead to a sense of belonging to the profession and explain their desire to care for infected patients.

“Despite all these circumstances, I want to choose nursing again because I truly like it and because I am interested in my profession, it really doesn’t matter what the patient is, whether it is infectious or trauma” (participant F).

### Accepting the nature of the job

Awareness of the dangers of the disease, acceptance of job hazards and job compatibility showed nurses accept the nature of the job. Familiarity with the risk of caring for an infectious patient from the time of education and awareness of the risk of infection transmission in all departments showed that nurses are aware of the risks of infectious diseases.

“Because I accepted this job, naturally I accepted all these hardships and I accepted the possibility of the dangers that may happen to me and the diseases that may occur as a result”. (Participant T)

### Professional ethics

Professional ethics are usually shown in official codes and include norms, obligations and perceptions of relationships between colleagues and the public. In the field of nursing, professional ethics may have a different meaning, it may refer to the personal characteristics of nurses, their virtues or manners and the correct manner of behavior. In addition to individual behavior, professional ethics now focuses on groups of professions that are guided by shared ethical codes [24].

Professional ethics in nurses refers to professional commitment, professional values and understanding the patient’s situation.

### Professional commitment

According to the statements of the nurses, responsibility, conscientiousness and sincere performance formed the main dimensions of professional commitment.

“I always carry on my work, even if they tell me that I won’t get paid for a month. I will still do my work as usual, even if they tell me I can do it however I like. Nothing can stop me from caring of patients”. (Participant L)

### Professional values

From the nurses’ point of view, work conscience, love for one’s fellow man, sacrifice and being supporter were important in the professional values and intention to care for patients with infectious diseases.

“I always worked compassionately and I tried to always work conscientiously” (Participant S).

### Understanding the patient’s situation

Nurses expressed sympathy, attention to the patient’s needs and relieve it, understanding the patient’s situation and the issue, can be one of the explanatory factors of a nurse’s intention to care for patients with infectious diseases.

“But when the patient was not getting better, I was very upset. anyway, someone else’s dear, just like our family is dear to us” (Participant H).

### Individual values

Individual values ​​mean comperhensive, necessary and extensive goals and are the basis of attitudes and behavior [25]. There is a lot of proof in Western societies that spirituality and religion are linked to better mental health. Previous studies have also demonstrated a beneficial connection between spirituality, religion, and mental health in Iran and other Muslim nations. Spirituality, religiosity, and self-esteem have also shown a positive correlation with psychological well-being in western cultures. Self-esteem and spirituality are intimately intertwined in Iranian culture, which can be seen by carefully examining it [26]. personal values in nurses refer to spirituality and self-esteem.

### Spirituality

Nurses stated that patients’ prayers and God’s satisfaction are very important to them and faith in God is very effective in the care of patients.

“The thing that has increased my motivation is the patient’s prayer and God’s satisfaction”. (Participant U)

### Self-esteem

The sense of worth and effectiveness in society, feeling good about the patient’s recovery and enjoying the care were the things that the nurses said. Additionally, they claimed that taking care of patients with infectious disorders has raised their degree of expertise in the field.

“I will try my best to help every person, whether young or old, just being able to make someone happy, in my opinion, can be one of the highest actions of a human being” (Participant A).

### Standard precautions

For managing healthcare-associated infections and lowering occupational health hazards, standard precautions are regarded as the fundamental preventative measures [27]. When nurses offer nursing care to patients, they may utilize needles that may be infected with a variety of infectious microorganisms and are exposed to patient blood or bodily fluids. This might make infections more likely. Thus, it is crucial for nurses to be aware of and follow recommended precautions in order to lower the risk of secondary infections [28]. In this study, Based on the opinions of nurses, Standard precautions refer to occupational safety and preventive measures.

### Occupational safety

Based on the opinions of nurses, the presence of personal protective equipment, sufficient equipment and the standard physical structure of the infectious ward is very effective in increasing their intention to care of infectious patients.

“Personal protective equipment is one of my priorities. First, life must be safe, then the property has value” (Participant N).

### Preventive measures

Nurses stated that compliance with the principles of control and prevention, the use of personal protective equipment and constant caution in caring for patients prevent the spread of infectious disease to them and can play a role in their intention to care for patients with infectious disease.

“We must comply with the care of all patients, for example, when suctioning patients, we must have gloves and all personal protective equipment, or even when we touch the patient’s bed sheet, it may be infected, so we must always comply” (Participant O).

### Preserving health

According to the World Health Organization, health is a comprehensive condition of mental, physical, and social well-being and does not just refer to the absence of sickness or disability [29]. According to the findings of earlier research conducted during the SARS and Ebola epidemics, labeling of health care personnel who have detrimental psychological disorders like worry, fear, or stress can have a significant negative impact on the caliber of their job and services. Contrarily, medical professionals are required to wear bulky protective clothing and masks, which limits their range of motion and makes it more challenging to carry out medical treatments and practices than under normal circumstances. All of these elements, along with the possibility of contracting an illness and spreading it to others, raise the possibility of psychiatric disorders in healthcare professionals [30].

In care of infectious patients, one of the most important concerns of nurses was to try to maintain their health and that of their families against the transmission of infectious diseases, as well as reducing physical and mental pressure.

### Health anxiety

The fear of contracting the sickness and transferring it on to their families and themselves, according to nurses, has made them less willing to care for patients with infectious disorders. Some experienced nurses stated that despite the fear of contagion, they can manage this fear.

“At heart, the feeling of anxiety and fear makes me avoid working with these patients”. (Participant I)

## Recognition of illness

According to the nurses’ opinion, lack of understanding of the disease’s nature, unknown diagnosis and known diagnosis can affect their intention to care of infectious patients.

“If the patient’s diagnosis is clear, one’s duty is clear, and you wear and observe personal protective equipment and go over the patient’s head” (Participant H).

### Prioritizing personal and family health

The nurses stated that the importance of individual and family health, the importance of the health of the vulnerable person in a family and special family circumstances reduce their intention to care for infectious patients.

“If it is according to the basic charts and I can take care of myself in the first place, I am willing, but if I want to make sacrifices and go over the head of this patient without anything, I will not do this” (Participant D).

### Physical and mental stress

Based on the point of view of nurses, physical and mental stress and exhaustion leads to a decrease in their intention to care for patients with infectious disease.

“The concern of working with these patients is more than other patients, and because you have this concern, it takes more energy from you and makes you more tired” (Participant N).

### Support

Reward and appreciation are strong predictors of job satisfaction and passion for nurses. In addition, nursing managers support nurses by rewarding them, increasing their interaction and facilitating the employment of nurses. Additionally, recognizing nurses’ contributions by nursing leaders improves patient care’s safety and quality. In addition, feeling recognized, appreciated, and respected increases nurses’ satisfaction with their rewards and decreases their willingness to leave. Effective use of reward methods can also reduce nurse turnover [31]. Incentives, both monetary and non-monetary, are crucial in deciding how well employees perform [32]. In this study, based on the opinions of nurses, support refer to financial support and non-financial support.

#### Financial support

According to nurses, financial incentives and salary increases encourage them to care for patients who are contagious, whereas low wages discourage them from doing it.

“Honestly, if we are well supported financially, the nurse’s enthusiasm for work will increase. When our income is much lower compared to other fields, we will be fired because the salary we get is much less compared to our job, this makes the passion we have for our work decrease day by day” (Participant O).

## Non-financial support

Nurses stated that the support of family, colleagues and head nurses as well as increasing the number of nurses, educational support, recruitment of nurses and media support can have a great effect on increasing The motivation for providing care to patients with infectious disorders.

“If the head nurse gives a good monthly nursing program to the nurses and respects the nurses’ holidays and gives a fair program, this is considered a type of support that can be effective” (Participant N).

## Attitude of patients and their families

Attitudes are a subject that includes an object, person, or an abstract idea. Attitudes towards others are in the field of interpersonal interest, attitudes towards oneself are in the field of self-confidence, and attitudes towards abstract ideas are in the field of values [33]. Reflecting the opinions of patients in the performance of a health organization improves the management of services and the behavior of health professionals in order to determine the appropriate management policies and procedures and prioritize the allocation of resources and training needs. National and international health service evaluation organizations determine patient satisfaction as one of the key indicators for controlling the results of health services and reimbursement of hospital costs [34]. According to nurses, the attitude of patients and their families included behavioral reflection and patient and their family satisfaction.

### Behavioral reflection

Based on the nurses’ point of view, appropriate or inappropriate behavioral responses of the patient and the behavioral reaction of the patient’s family can affect their motivation to care for patients.

“A good response from the patient gives me energy and motivates me, and I do my work with more enthusiasm” (Participant M).

### Patient and family satisfaction

Based on the nurses’ point of view, understanding the patient from the nurse, patients and their family satisfaction increases the motivation to care for infectious patients.

“Especially when a patient is conscious, she recognizes and thanks you or the patient’s family feels satisfied somehow, I get a very good feeling” (Participant E).

## Discussion

The present study explains the concept of nurses’ intention to care of patients with infectious disease among Iranian nurses. Based on data analysis seven main categories extracted, which included job satisfaction, compliance with professional ethics, personal values, standard precautions, maintaining health, support and attitudes of patients and their families. Job satisfaction was one of the dimensions of the concept of nurses’ intention to care of patients with infectious disease. Human resources are key elements to improve the performance of the healthcare system and a sufficient number of competent and motivated healthcare workers are needed to achieve national and international goals related to health [35]. The primary element influencing nurses’ perseverance at work is job satisfaction [23]. Recent research has demonstrated the importance of job satisfaction for ensuring the standard of patient care and nurses’ intention to care [36]. According to the findings of the study by Sharif Nia et al., job satisfaction and organizational commitment are variables that help to understand the relationship between nurses’ workload and their intention to provide care to COVID-19 patients [9]. In current study, the dimension of job satisfaction was one of the main dimensions explaining the intention of nurses to care for infectious patients.

Professional ethics was the second dimension of the concept of nurses’ intention to care for infectious patients, which is explained in current study. Professional ethics relies on individual commitment and responsibility in the role of a nurse. Compliance with professional ethics is very effective in nurses’ ethical decision-making and responding to the challenges of current changes in health care [24]. In Lam’s study, nurses’ professional attitudes, which included the categories of a sense of commitment and professional spirit, were one of the primary categories derived from emergency nurses’ knowledge of their professional responsibilities during the influenza pandemic [37]. In current study, the main category included professional commitment and professional values, which are in line with Lam’s study. In the study of Sadati et al., one of the main categories extracted from nurses’ percept and experiences of the outbreak of COVID-19 was a selfless commitment [16]. These results show that the sense of responsibility, conscientiousness, and sacrifice, which are the principles of professional ethics [38], are important factors that explain nurses’ perception of the intention to care for patients with infectious diseases.

The third dimension of the concept of nurses’ intention to care of infectious patients was personal values which are the basis of attitudes and behavior [25]. Spirituality and self-esteem formed the main category of this main category. A thorough examination of Iranian culture reveals a connection between spirituality and self-esteem [26]. The results of our study showed that nurses felt valued and effective by caring for infectious patients, but in Muz and Erdogan study, one of the experiences of nurses in caring for patients with COVID-19 was the feeling of social isolation and loneliness [39], which was contrary to the results of current study. In the study of Alilu et al., spiritual satisfaction, efficient attendance and development of professional capabilities were effective barriers on nurses’ intention to leave clinical nursing [40], which is consistent with this study. These results show according to the culture of the society in Iran, spirituality and self-esteem are very effective in the intention of nurses to care for infectious patients.

From the data analysis, standard precautions that included occupational safety and preventive measures were also extracted. Standard precautions are necessary to manage healthcare that is related to infections and reduce occupational hazards [27]. According to the findings of the Said and Shafei study, nurses’ intention to quit their jobs during the COVID-19 disease epidemic was most strongly correlated with being exposed to the danger of infection [41]. In our study, nurses expressed that access to personal protective equipment and compliance with the principles of control and prevention are effective factors in the intention to care for patients with infectious diseases. In Koh’s study, avoiding contact with patients, and observing the use of personal protective equipment were identified as ways to control the infection and reduce the risk of transmission of respiratory infectious diseases [42]. These findings supported the idea that nurses’ perceptions of the danger of disease transmission can influence their decision care for patients with infectious disorders.Therefore, healthcare organizations must ensure appropriate protective measures to control infection and the adequacy of personal protective equipment to protect nurses.

Another aspect of the idea of nurses’ intention to care for infected patients that was drawn from the data analysis was preserving health. Given that caring has the characteristics of chronic stress, creates mental and physical pressure during long periods of time [43]. Additionally, caring for contagious patients raises the risk of medical staff developing psychiatric disorders due to the possibility of contracting the illness and spreading it to others [30]. In this regard, the nurses in the current study acknowledged their worry about the disease spreading to themselves and their families as well as the psychological and physical strain brought on by caring for patients who were contagious. Considering the preservation of their own health and even the family, they intent to care of infectious patients. Beyond fear and uncertainty was the key main category in Lee’s study, which looked into the experiences of Korean nurses caring for patients with Middle East Respiratory Syndrome [14]. In Lam’s study, concern about health was one of the principal main categories extracted from the data analysis [37]. Several studies have shown that caring for patients with infectious diseases puts nurses under increased physical and mental pressures [39, 44] as well as clinical stress[45] which is consistent with the findings of current study.These findings demonstrate that the conflict between professional responsibilities, transmission hazards for diseases, and physical and emotional stress is foreseeable. Therefore, it is very necessary to instruct nurses and regularly assess occupational stress, and use appropriate ways to reduce fear among nurses.

The next main category explaining the concept of nurses’ intention to care for patients with infectious diseases was supporting, which included financial support and non-financial support. Incentives, both financial and otherwise, play an important role in shaping the quality of employees’ performance [32].The impact of support on job satisfaction and caring intentions [46] and the desire to continue working as a nurse [47] have been discussed. Also, the intermediary role of social support and psychological support in the decision to transfer healthcare workers has been confirmed [48]. The findings of the present study demonstrated that the financial incentives and support of the family and hospital officials explained the intention of nurses to care for patients with infectious diseases.

Patient and family attitude along with two categories of behavioral reflection and patient and family satisfaction was the last dimension explaining nurses’ intention to care. The positive attitudes of patients and their families towards nurses increase the motivation of nurses to support and care for patients [49]. According to this study, the patient’s proper behavior and the patient’s and patient’s family’s satisfaction with the nurse are important elements in raising nurses’ motivation to provide care for patients.

### Relevance to clinical practice

If nurses are forcibly used in infectious wards in medical centers, it will cause mental damage and stress and anxiety and moral distress for them [9]. Un intention to care of these infectious patients will cause insufficient and compassionate care to be provided to the patients, and this issue will prolong the course of recovery and increase treatment costs and reduce the turnover of hospitalized patients. Increase intention to care for patients with infectious diseases, especially during the outbreak of infectious diseases, has increased the quality of care for these patients, and the managers of the medical system will not need to be forced to use and provide staff for these departments, and there will be less nursing leave. As a result, nursing managers can use the study’s findings to address the challenges that nurses have when caring for patients with infectious diseases, as well as to comprehend these patients’ requirements and the factors that may make them reluctant to get care.

## Conclusion

The concept of nurses’ intention to care of patients with infectious disease from the standpoint of nurses includes different dimensions. In order to satisfy the healthcare needs of patients with infectious diseases, especially during infectious disease outbreaks, adequate planning, policies, and guidelines must be implemented. Managers can act by examining the problems and demands of nurses and creating job security to establish a safe healthcare system for an effective response during the outbreak of infectious diseases. Managers can also prevent leaving clinical nursing by providing suitable activities for nurses and increasing their motivation and satisfaction. In addition, it is necessary to develop psychosocial support to protect nurses from severe physical and psychological stress.

### Limitations

The time of current study was during the epidemy of Covid-19, due to the relevant restrictions, it was difficult for the interviewer to attend the hospital. Due to the working conditions of nurses in the infectious wards and the use of masks, gloves and gowns in the department, the interview conditions were difficult. The time limitation of the participants in the study was one of the other limitations of the study. To remove these limitations the research team chose the appropriate time based on coordination with the participants and head nurses and also invited the participants to be interviewed in their free time.

### Electronic supplementary material

Below is the link to the electronic supplementary material.

Supplementary Material 1

## Data Availability

Due to university policies, the datasets generated and utilized for the present study are not publically accessible but are available from the corresponding author upon justifiable request.
