# Supplementary material for: Nurses’ intention to care for patients with infectious disease: a content analysis study
Source: BMC Nurs. 2023 Oct 3;22:349. doi: 10.1186/s12912-023-01538-9 (PMC10548695; doi:10.1186/s12912-023-01538-9)
Supplement: Supplementary file 1 — Supplementary Material 1 [file 12912_2023_1538_MOESM1_ESM.docx]

**Interview 20 Mrs. ...**

19 years of work experience - official employment - married - no children - ICU ward - 5 Azar - 42 years old

**Researcher:** What made you want to take care of infectious patients?

**Participant:** After all, anyone who studies nursing, from the time they are students and work as trainees, and they work with all the patients. Of course, the job that one chooses checks all the criteria, and the possibility of danger may even occur in non-infectious departments, because I accepted this job, of course. I accepted all these hardships and I accepted the possibility of the dangers that may happen to me and the diseases that may occur as a result.

**Researcher:** How did you feel about these patients before you encountered them and how do you feel now?

**Participant:** Well, there are a series of protocols for our own protection and safety, which we try to follow. Actually, I don't have the personality to be afraid of diseases, but every human being has a fear of infectious diseases, and because I am married and have a family, even if it is not for me, this disease may spread to someone who does not want it at all. . There is a small amount of fear against these diseases, but I try to reduce this anxiety by following the protocols Koch taught us. At the beginning of the work, because the clinical experience is less, we do not have to deal with different patients during our student days, then we see the complications of those diseases and the events that happen to that patient, and these are not without the effects that the experience may have. It will reduce a series of anxieties.

**Researcher:** Did you think about the risk of disease transmission during this working period?

**Participant:** I said that because you accepted our job and we accepted this responsibility, we must be responsible for these diseases. And the fact that I, as a nurse, cannot separate these patients and say that you must give me the care of a series of patients who are not infected at all, or I can choose a department that may be a series of patients who are not aware of it themselves and under They have been hospitalized for another disease because we cannot isolate and I accepted this responsibility, naturally I have to do this work.

**Researcher:** What is your opinion about the lack of benefits of working in these departments compared to non-infectious departments?

**Participant:** Financially, it doesn't matter to me, and even heart patients may have their own difficulties, and we cannot separate and say infectious patients and those who work in the infectious department, considering that there is contagion and there is a possibility of danger. And the person who works in the oncology department also has the risk of complications from the chemotherapy of the patients, that's why we can't separate them, and each department has its own characteristics, and financially, we can't say that the person who works in the infectious department should be paid in terms of salary. and receive more benefits.

**Researcher:** I mean, do you have the same opinion even if you work in a purely infectious department such as respiratory isolation?

**Participant:**  You see, not financially, but feedback who is working in a department, by nature, that person's morale is also affected and he may suffer from chronic fatigue, and that he has to wear that personal protective equipment all the time, even in hot weather. A place that may not have proper ventilation naturally affects the mood and causes chronic fatigue, and this affects the personal life of the person, so I expect that my shifts will be less and I will have more vacations so that energy can be renewed.

**Researcher:** In your opinion, what other conditions should be provided for you?

**Participant:** The number of patients should be less and the number of personnel should be enough so that I don't have to come to the Long Guards in the evening or in the morning, because the night guard alone has its own fatigue and the number of guards given to one person and the number of personnel. And the number of patients that are given to the nurse and the holidays that are considered for them, the incentives, even if it is not financial, it is a motivation for the person, financially, if there are circumstances, it is much better to thank the staff from time to time. And if not, non-financial incentives are also effective.

**Researcher:** What do you think about the support of the authorities?

**Participant:** The support of the supervisor is also very important when you are taking care of infectious patients, vegan and wearing gloves, and at any moment you want to put something on paper and document it, and if he wants to document it at the end, he might forget something. Documenting, it is possible to forget something that does not even harm the patient's life, the supervisor should not highlight that error, which will cause the person to lose motivation.

**Researcher:** Do you mean that the guardian's support has a great impact on your intention to care?

**Participant:**  Both the supervisor and the colleagues of the department, for example, when a patient is seriously ill, it is necessary that the colleague you are working with should really understand him and help you. Or maybe I'm on duty, but I'm not in good general condition, I have a fever or other symptoms. When I call the nursing office or the supervisor, they should trust my words, they shouldn't say that he wants to bring false illness or he wants to go for fun. Personnel and officials must have the necessary honesty to be able to do this cooperation.

**Researcher:** What do you think about family support considering that you are both a wife and a daughter of the same family?

**Participant:** First of all, there is a family of parents who know their children's mentality and have no problem with this, and in the case of my wife, it can be said that they measure my conditions and accept all the problems of my work, and then marriage takes place. Therefore, there must be family support, and if there is not, all the pressure will be placed on that person, and it will certainly not be bearable.

**Researcher:** I mean, what if your husband said that she doesn't like you to work in the infectious department?

**Participant:** You see, there is always department rotation in our work, and sometimes, like during the outbreak of Corona, there are patients with Corona in all hospitals, so I will discuss all this with my wife at the time of marriage and tell her that I may be placed in any department due to the rotation, and explain my work shifts to her. After 18 years of work, I am still doing night duty and I still go on vacation and I have no problem with my wife and family. I just have to take a series of precautions that are necessary and I am taking care of an infectious patient so that it does not spread to the family. Because like corona disease, I may be asymptomatic, but I may be a carrier and I must be careful not to pass it on to my parents who may have an underlying disease. We should control the family and reduce the risk of transmission by following the protocols, but it is not true to say that I am afraid of spreading the disease to the family and I do not work in the infectious department.

**Researcher:** What do you think about personal protective equipment and how much can it affect your intention to care?

**Participant:** It is very effective, for example, if you think it is a patient with hepatitis or a respiratory disease whose secretions I work with, well, if I do not wear eye shields or protective glasses, the secretions may spread and cause the disease to spread to me, if I use any of this personal protective equipment. I should not use it and say that it is difficult to wear these, so the risk of disease transmission may increase

**Researcher:** So what if you lack personal protective equipment in the department?

**Participant:** Some devices like the gun can be used in one operation, and if there is no mask, I can wear several layers of masks or, for example, sterile gloves, I can wear several layers of latex gloves on top of each other. Personal protection in their consumption managed

**Researcher:** Do you mean that the lack of personal protective equipment can't affect your intention to care?

**Participant:** No, you can't, you have to manage when there is a shortage, like in your own house, when sometimes a guest comes and you don't have enough catering equipment and you have to manage.

**Researcher:** Can the experience you gain in caring for an infectious patient affect your intention?

**Participant:** Yes, of course, the experience we have gained over the years in clinical work is not without influence, for example, I have worked with corona patients and we have seen that when it happens to my family or relatives, I can guide them much more easily. We can talk more strongly about the disease and give our opinion.

**Researcher:** If you are not aware of the infectiousness of your disease, you tend to take more care Do you know?

**Participant:** Of course, it's better to know, because if I don't know, I might deal with him like a normal patient and not use personal protective equipment completely, but when I know that the patient is infectious, the number of times I go over the patient and wear personal protective equipment is different, and it is certainly much better to know that I am an infectious patient. LT is.

**Researcher:** There are a series of infectious patients who either don't know about the disease or if they do, they don't tell you. What do you think about the care of these patients?

**Participant:** Yes, some patients such as HIV or hepatitis do not announce their disease in order not to be rejected from the society. Regarding these, we in nursing should assume that all patients may have an infectious disease such as HIV and Even they don't know or they know and try to hide it, so we have to use a series of personal protection devices to reduce this risk.

**Researcher:** Do you mean that we must observe the principles of personal protection at all times and conditions?

**Participant:** Yes, we should always wash our hands and wear gloves, which means we should always be ready.

**Researcher:** When working in an infectious department, how much can it affect your intention to care if the colleagues of the department do not follow the principles of personal protection or cause the disease to spread with their actions?

**Participant:** I might get annoyed because it is a team work and the rest room is for one person and for example you want to have tea, we are all together and we are spending a few hours together in the ward. Finally, the risk of disease transmission due to non-compliance threatens my colleague, so in the intention to My care makes an impact.

**Researcher:** Can the experiences of colleagues affect your intention?

**Participant:** Of course, usually at the time of starting work, there are more stresses and fears, and when there is a long-term colleague, when something happens to you or during clinical work, he is by your side and he is supporting you, and this has an impact on your intention.

**Researcher:** The conclusion of the treatment can also affect the intention to care.

**Participant:** Of course, when a patient gets better, he gets energy from his work, but when you work a lot on a patient and something might happen all of a sudden and the insurance expires, this is a negative feedback. But if the doctor's order is appropriate and the diagnosis is correct, and the colleagues work properly, and the patient himself and the patient's companion have given the necessary cooperation, all this is a positive feedback that makes one want to take care of the patient more.

**Researcher:** What do you think about the behavior of the patient or the patient's companion during care and how much does it affect your intention to care?

**Participant:** In hospitalized patients, especially infectious patients, because they are hospitalized for a long time, they may develop a series of mood disorders and their behavior becomes bad, and it is not that I do not want to take care of them because their behavior is inappropriate. And we do not say that they should be grateful for the patient's behavior, but the fact that it does not interfere with our work does not have an effect on the intention to care. But it is not to say that the accompanying behavior makes me not take care of the patient.

**Researcher:** If the patient is infected, do you still want to take care of this patient?

**Participant:** In nursing, we have something called ethics in nursing. For this patient, we must provide them with a series of support, such as palliative care. Complete

**Researcher:** How much energy does work with infectious patients take from you?

**Participant:** Of course, it takes more energy from me and even affects my mood because we are always worried about where the treatment of this patient will lead and whether we will get a positive result or not, and the patient's companion or the patient himself will constantly ask you.

**Researcher:** And do these issues affect your intention to care?

**Participant:** When you are spending so much energy and you are taking care, for example, the treatment of the supervisor or the management treatment, for example, the nursing office, if the necessary support is not provided and long shifts are given and leave is not given and proper support is not provided, no matter how much you have a heart intention to care If you have these patients, after a while, this fatigue becomes so negative that you become reluctant to care.

**Researcher:** How much does the physical problems caused by taking care of infectious patients affect your intention to take care?

**Participant:** Excessive use of masks causes breathing problems, and the use of hand wraps and gloves causes skin problems. In the long term, these will reduce the intention to care, so it is necessary to change the department at intervals of rotation and long years of working in This department or even other departments such as the ICU, which is a heavy work, causes fatigue and reduces the intention to care.

**Researcher:** How is your inner belief about the intention to care?

**Participant:**  We have a series of religious beliefs and we have a series of personal beliefs, such as when a patient is treated and we see the patient's happiness, it gives you positive energy and makes you work with more energy for another patient, and in terms of your religious belief, this care And nursing a person who needs you to take care of him at this time so that he can pass this stage, I think it gives a good feeling.

**Researcher:** Thank you for your time.
